# A risk score model based on endoplasmic reticulum stress related genes for predicting prognostic value of osteosarcoma

**DOI:** 10.1186/s12891-023-06629-x

**Published:** 2023-06-23

**Authors:** Yong Zhao, Jijian Gao, Yong Fan, Hongyu Xu, Yun Wang, Pengjie Yao

**Affiliations:** Department of Orthopaedic Surgery, Shengzhou People’s Hospital (the First Affiliated Hospital of Zhejiang University Shengzhou Branch), Shaoxing, 312400 Zhejiang China

**Keywords:** Endoplasmic reticulum stress, Osteosarcoma, Overall survival, Survival prediction model

## Abstract

**Background:**

We aimed to establish an osteosarcoma prognosis prediction model based on a signature of endoplasmic reticulum stress-related genes.

**Methods:**

Differentially expressed genes (DEGs) between osteosarcoma with and without metastasis from The Cancer Genome Atlas (TCGA) database were mapped to ERS genes retrieved from Gene Set Enrichment Analysis to select endoplasmic reticulum stress-related DEGs. Subsequently, we constructed a risk score model based on survival-related endoplasmic reticulum stress DEGs and a nomogram of independent survival prognostic factors. Based on the median risk score, we stratified the samples into high- and low-risk groups. The ability of the model was assessed by Kaplan–Meier, receiver operating characteristic curve, and functional analyses. Additionally, the expression of the identified prognostic endoplasmic reticulum stress-related DEGs was verified using real-time quantitative PCR (RT-qPCR).

**Results:**

In total, 41 endoplasmic reticulum stress-related DEGs were identified in patients with osteosarcoma with metastasis. A risk score model consisting of six prognostic endoplasmic reticulum stress-related DEGs (*ATP2A3, ERMP1, FBXO6, ITPR1, NFE2L2*, and *USP13*) was established, and the Kaplan–Meier and receiver operating characteristic curves validated their performance in the training and validation datasets. Age, tumor metastasis, and the risk score model were demonstrated to be independent prognostic clinical factors for osteosarcoma and were used to establish a nomogram survival model. The nomogram model showed similar performance of one, three, and five year-survival rate to the actual survival rates. Nine immune cell types in the high-risk group were found to be significantly different from those in the low-risk group. These survival-related genes were significantly enriched in nine Kyoto Encyclopedia of Genes and Genomes pathways, including cell adhesion molecule cascades, and chemokine signaling pathways. Further, RT-qPCR results demonstrated that the consistency rate of bioinformatics analysis was approximately 83.33%, suggesting the relatively high reliability of the bioinformatics analysis.

**Conclusion:**

We established an osteosarcoma prediction model based on six prognostic endoplasmic reticulum stress-related DEGs that could be helpful in directing personalized treatment.

## Background

Osteosarcoma (OS) is the most common primary bone tumor that commonly arises in the osteogenic skeletons within membranes in both children and young adults [1]. OS tumors grow rapidly during the early stage of the disease, which is followed by systemic dissemination. Although the prognosis of OS has improved through the combination of chemotherapy and surgery, the long-term survival rate remains unsatisfactory in > 30% of patients [2]. Thus, it is challenging to improve the survival of patients with OS, and novel diagnostic approaches and therapeutic strategies are urgently needed for the same.

With the increasing knowledge of molecular profiling and the creation of robust model systems, various genetic alterations have been detected in OS. The endoplasmic reticulum (ER) is the first metabolic compartment for several biochemical processes and reactions [3]. The ER mainly plays is the organelle in which secretory protein and membrane synthesis occurs, where proteins fold into their native conformations [4, 5], intracellular Ca2 + is stored, and lipids and sterol biosynthesis occurs. Multiple studies have supported the role of ER stress (ERS) in OS. For example, Shimizu et al. demonstrated that the therapeutic role of calcitriol in OS was mediated through ERS via the downregulation of cyclin D1, activation of intracellular production of reactive oxygen species, and activation of the p38 MAPK pathway [6]. Additionally, ERS has been reported to mediate apoptosis in human OS [7, 8]. Zhao et al. reported that β-elemonic acid induces ERS-mediated suppression of the Wnt/β-catenin pathway and activation of the PERK/eIF2α/ATF4/CHOP axis in OS [9]. Taken together, these findings suggest that ERS-related genes may be promising molecules for predicting the effectiveness of ERS-based treatment approaches. However, the gene signatures related to ERS in the prognosis of OS and associated metastasis need to be explored further.

Therefore, in the present study, to improve the diagnosis of OS, we attempted to construct an OS prognostic prediction model based on the signature of ERS-related genes. First, OS-associated RNA sequencing datasets were downloaded and differentially expressed genes (DEGs) between OS with and without metastasis were identified. Next, candidate prognostic ERS-related genes were screened, and a risk score (RS) model based on the identified prognostic ERS-related genes and a nomogram survival model of independent survival prognostic factors were constructed. The ability of the model was assessed by Kaplan–Meier (KM) analysis and a receiver operating characteristic (ROC) curve. Our results contribute to the understanding of the pathogenesis and progression of OS metastasis with respect to ERS.

**Table 1:** The sequences of all primers

Primer	Sequence (5’-3’)
GAPDH	F: AGACAGCCGCATCTTCTTGT
	R: CTTGCCGTGGGTAGAGTCAT
FBXO6	F: TGGAAAATCTTCTACTTCCTACGG
	R: AATACTTCTTGACTTTGGGGTCAG
NFE2L2	F: TTGACATACTTTGGAGGCAAGATA
	R: GTGACTGAGCCTGATTAGTAGCAA
ITPR1	F: TGTCTACACAGAGATCAAGTGCAA
	R: CCACATGTGATTGCTGGTATAAAT
ERMP1	F: AATTTCTCACATAACCCCTCACAT
	R: CAAGGTGTCTGTTCTTTGGATATG
USP13	F: TGACGATTTAAATAGCGACGATTA
	R: GTCCTGCTTTCTGTATGGAGATTT
ATP2A3	F: TCCTTTAACGAGATCACTGCTATG
	R: GAATTGCTTCATGTTGCTGTAGAT

## Methods

### Datasets and preprocessing

The gene expression profile of OS-related RNA-seq data downloaded from UCSC Xena (https://xena.ucsc.edu/) was based on the Illumina HiSeq 2000 and used as the training dataset. After corresponding the clinical information of the sample, 176 of the 265 OS samples in the dataset were included in the present study.

Additionally, the dataset GSE39055 was downloaded from the NCBI GEO database, and it was based on the Illumina HumanHT-12 WG-DASL V4.0 R2 expression beadChip platform [10, 11]. This dataset included 37 OS samples, of which 36 samples had corresponding clinical survival and prognostic information and were used as the validation dataset.

**Fig. 1:**
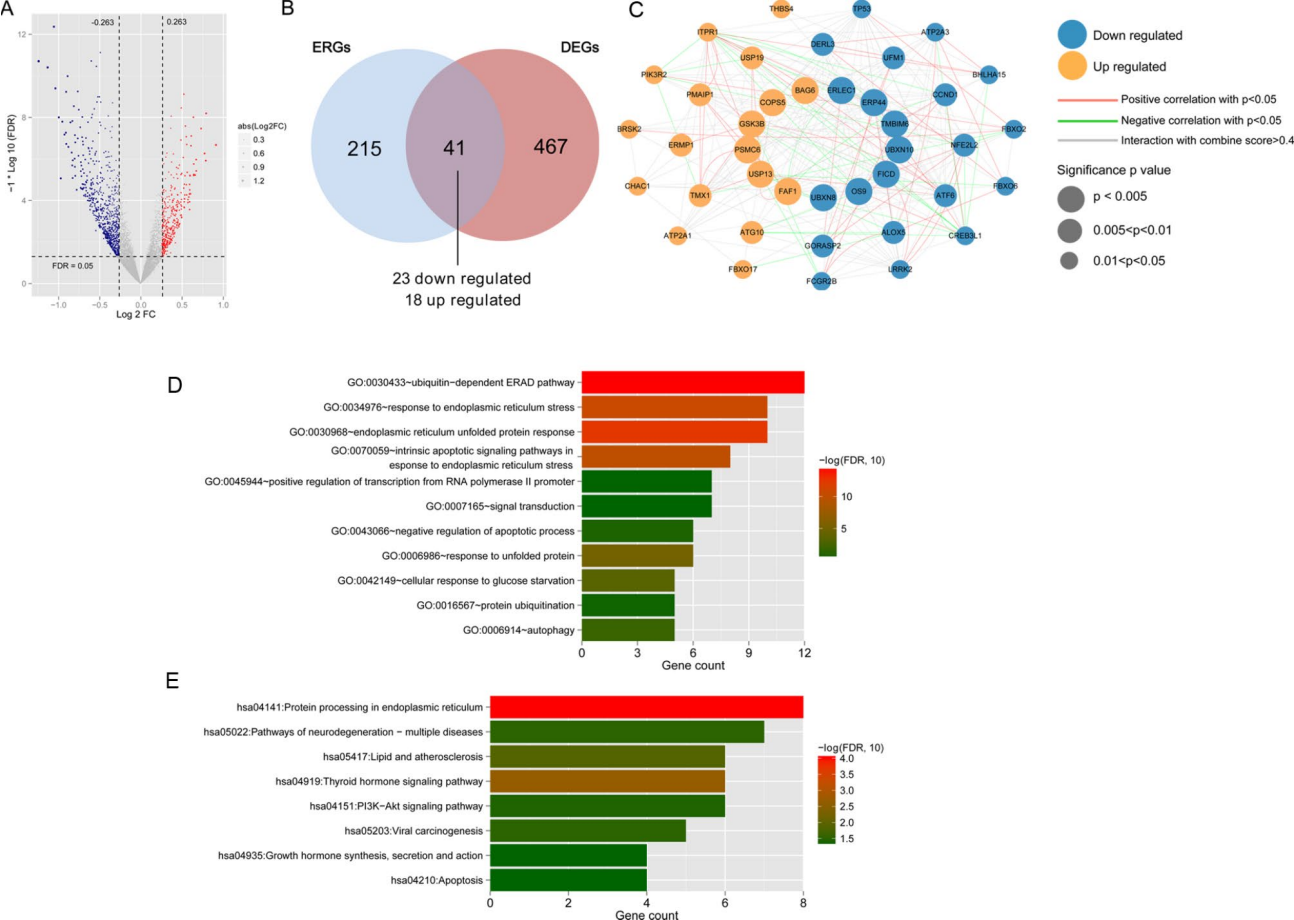
Differentially expressed genes (DEGs) in patients with osteosarcoma and overlapping endoplasmic reticulum stress (ERS)-related genes. a: Volcano map of DEGs in patients with osteosarcoma with and without metastasis. Red and blue dots indicate significantly upregulated and downregulated DEGs, respectively; horizontal dotted lines indicate false discovery rate < 0.05; and the two vertical dotted lines indicate | log2fold change| > 0.263. b: Wayne Chart of ERS-related genes and DEGs. c: The protein–protein interaction network of ERS-related DEGs. Blue and orange nodes represent downregulated and upregulated genes in the transferred samples, respectively. The size of the node indicates the significance of the difference, and the larger the node represents the higher significance. Red and green connections represent significant positive and negative correlation, respectively, while gray connections represent interactions. d: Column diagram of biological process. e: Column diagram of Kyoto Encyclopedia of Genes and Genomes (KEGG) pathway (www.kegg.jp/kegg/kegg1.html, Kanehisa Laboratories, Kyoto, Japan). The horizontal axis represents the number of genes, the vertical axis represents the item name, and the column color represents significance

### ERS-related differentially expressed genes (DEGs)

OS samples in The Cancer Genome Atlas (TCGA) were divided into two groups: with and without metastasis. Subsequently, the limma package (version 3.34.7) [12] in the R3.6.1 was used to screen DEGs between the two groups. The thresholds were defined as a false discovery rate (FDR) < 0.05, and |log_2_ fold change (FC)| > 0.263.

Subsequently, based on the MSigDB module in the Gene Set Enrichment Analysis (GSEA) database, the genes related to “GOBP RESPONSE TO ENDOPLASMIC RETICULUM STRESS” and “GOBP REGULATION OF RESPONSE TO ENDOPLASMIC RETICULUM STRESS” were downloaded [13]. After comparison with the screened DEGs, the overlapping genes were selected as ERS-related DEGs for further analysis.

**Fig. 2:**
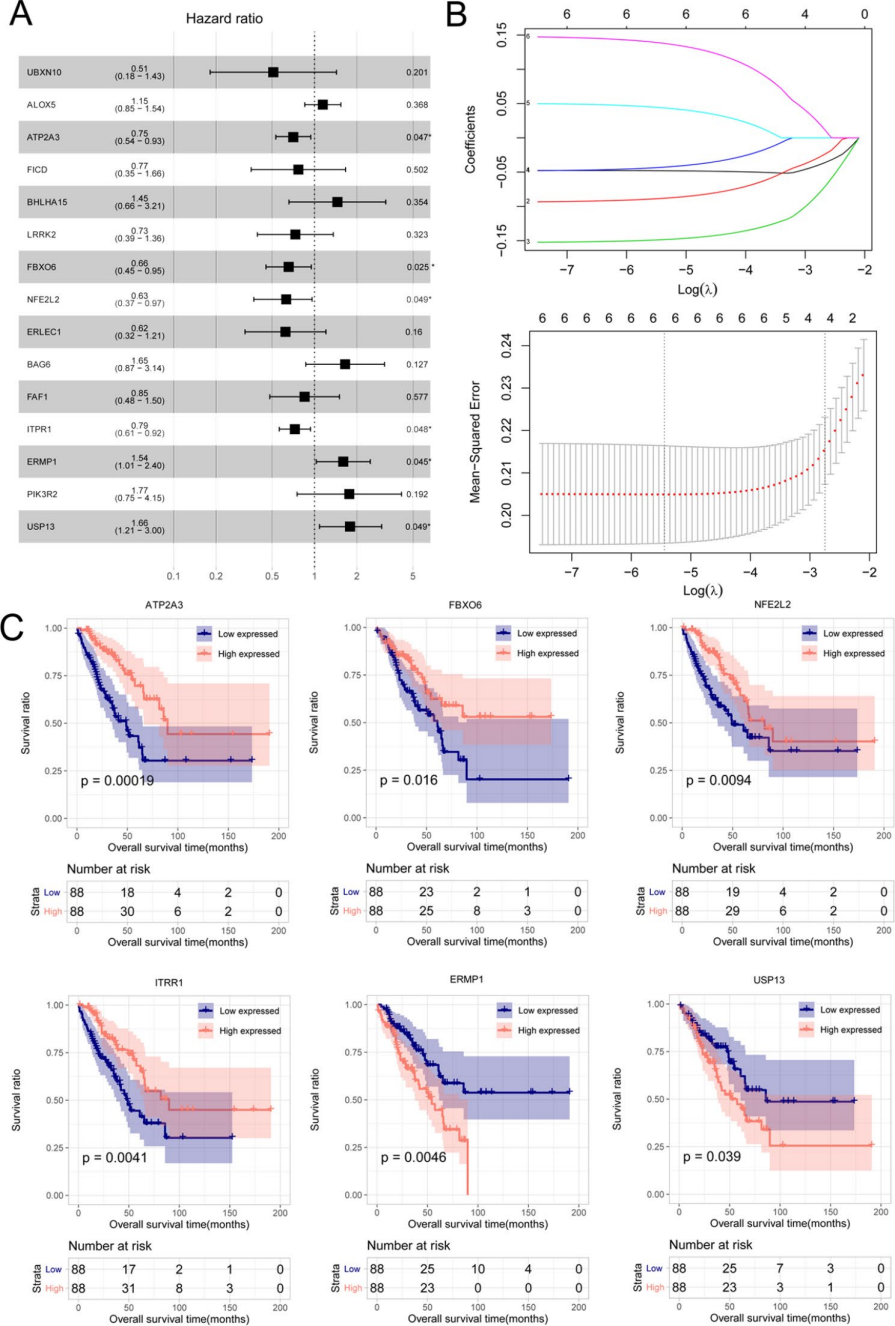
Forest map of prognostic ERS-related genes, LASSO parameter diagram, and prognostic Kaplan-Meier (KM) curves. a: Forest map of ERS related genes significantly related to survival. b: LASSO parameter diagram. c: Prognostic KM curves of six optimized survival related ERS genes. Blue and red curves represent the groups with low and high level of expression, respectively

### Construction of the protein–protein interaction network

The relationship between the protein products of the identified ERS-related DEGs was analyzed using the STRING database (Version 11.0) [14], and protein pairs were included when the interaction score was higher than 0.4. Based on the expression of the selected ERS-related DEGs, the Pearson correlation coefficient (PCC) was calculated using the cor. function in R3.6.1. Co-expression relationship pairs with p values less than 0.05 and absolute PCC values higher than 0.3 were defined as significantly related protein–protein interaction pairs. We constructed and visualized the integrated network using Cytoscape (version 3.6.1) software [15]. Subsequently, enrichment analysis was performed using the Kyoto Encyclopedia of Genes and Genomes (KEGG, approved by Kanehisa laboratories, Kyoto, Japan) signaling pathway [16–18] and gene ontology (GO) of biological processes to investigate the potential functional enrichment using the Database for Annotation, Visualization, and Integrated Discovery (DAVID, Version 6.8) [19, 20], and p-values less than 0.05 was defined as statistically significant.

**Fig. 3:**
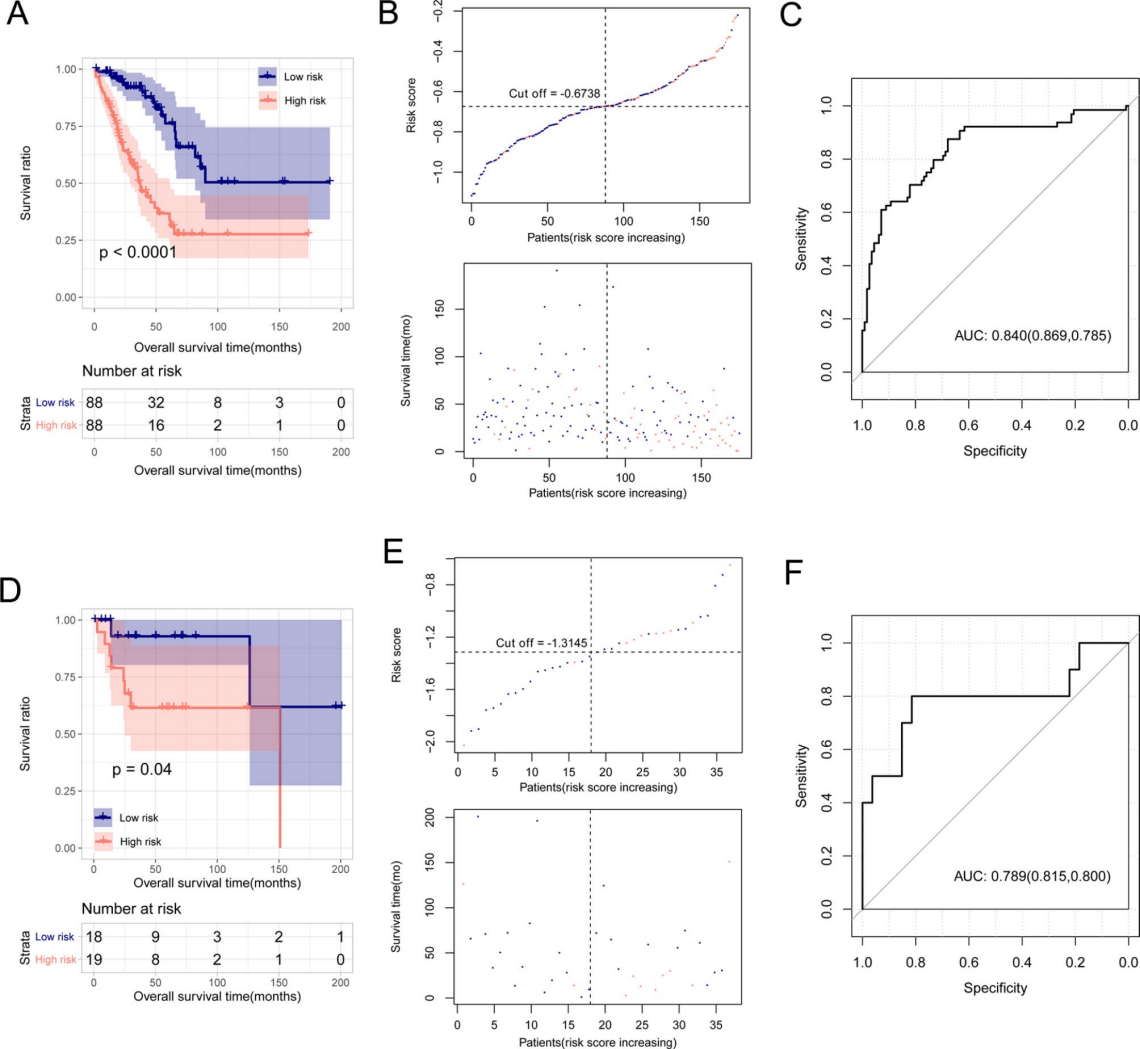
KM curves, risk score distribution, and ROC curves of prognosis models based on ERS-related genes. a: KM curves based on the six optimized ERS-related genes in the TCGA training set. Blue and red curves represent low- and high-risk groups, respectively. b: Risk score and the status of survival time based on the six optimized ERS-related genes in the TCGA training set. c: ROC curves based on the six optimized ERS-related genes in the TCGA training set. The numbers in brackets represent the specificity and sensitivity of the corresponding ROC curves. d: KM curves based on the six optimized ERS related genes in the GSE39055 dataset. Blue and red curves represent low- and high-risk groups, respectively. e: Risk score and the status of survival time based on the six optimized ERS-related genes in the GSE39055 data set. f: ROC curves based on the six optimized ERS-related genes in the GSE39055. The numbers in brackets represent the specificity and sensitivity of the corresponding ROC curves

### Construction of prognostic model of ERS-related genes

Based on the clinical survival prognosis information provided in the TCGA dataset, single-factor Cox regression analysis was performed [21] to screen the ERS-related genes that were associated with overall survival. Subsequently, multi-factor Cox regression analysis was performed to screen for ERS-related genes that were significantly correlated with prognosis. Gene sets with a p value less than 0.05 were considered as statistically significant. Finally, survival regression analysis was performed using the LASSO algorithm in the lars package of R3.6.1 (Version 1.2) to screen the optimal ERS-related genes [22]. Based on the LASSO prognostic coefficient of the optimized ERS-related genes and the level of expression of the target genes in the dataset, the RS model was constructed using the following formula: RS = ∑Coef_genes_ ×Exp _genes_; wherein, Coef_genes_ is defined as the LASSO prognosis coefficient of the target gene and Exp_genes_ is defined as the level of expression of the target gene.

**Table 2:** Clinical Prognostic Factors Prognostic Correlation Table

Clinical characteristics	TCGA(N = 176)	Uni-variables cox			Multi-variables cox		
		HR	95%CI	P	HR	95%CI	P
Age (years, mean ± sd)	61.10 ± 15.21	1.018	1.001–1.036	3.99E-02	1.01	1.003–1.309	4.70E-02
Gender (Male/Female)	72/104	1.06	0.642–1.750	8.20E-01	-	-	-
Pathologic tumor depth (mean ± sd)	6.35 ± 3.68	1.135	1.051–1.225	9.96E-04	1.02	0.871–1.196	8.04E-01
Pathologic tumor length (mean ± sd)	11.89 ± 7.25	1.062	1.031–1.093	3.91E-05	1.055	0.924–1.204	4.27E-01
Pathologic tumor width (mean ± sd)	8.85 ± 5.51	1.092	1.043–1.144	1.37E-04	0.999	0.845–1.183	9.97E-01
Tumor recurrence (Yes/No/-)	28/141/7	2.603	1.533–4.422	2.38E-04	1.162	0.515–2.623	7.17E-01
Tumor metastatic (Yes/No)	56/120	3.014	1.834–4.954	4.80E-06	2.969	1.471–5.996	2.40E-03
Radiotherapy (Yes/No/-)	64/110/2	0.865	0.517–1.447	5.80E-01	-	-	-
Tumor necrosis (No/Slight/Moderate/Severe/-)	61/35/59/11/10	1.182	0.923–1.513	1.83E-01	-	-	-
RS model status (High/ Low)	88/88	3.983	2.289–6.928	1.49E-07	4.851	2.258–10.423	5.19E-05

Subsequently, the RS values of the genes involved in the GSE39055 validation dataset and the TCGA training set were calculated. Based on the median RS values, we stratified the samples into high- and low-risk groups. The correlation between the high- and low-risk groups and the actual survival prognosis was evaluated using the Kaplan–Meier (KM) curve method in the survival package in R3.6.1 [22].

### Nomogram survival rate model construction

The distribution of clinical information in samples from the two risk groups was further analyzed. Clinical factors associated with independent survival prognosis were screened using the single-factor and multi-factor Cox regression analysis survival packages (Version 2.41-1) in R3.6.1 [22], and the clinical factors with log rank p value less than 0.05 were defined as independent survival prognosis-related factors. To evaluate the association between independent prognostic clinical factors and the RS model enrolled factors, one year, three year and five year-survival prediction models of the nomogram were evaluated using the rms package (version 5.1-2) in R3.6.1 [23, 24]. Subsequently, the C-index coefficient of the nomogram prognostic model was calculated using the survcomp package in R3.6.1 (Version 1.34.0). The C-index represented the score of all individual pairs correctly sorted based on Harrell’s statistics [24–26].

To analyze the net benefit of the survival-related factors and compare the impact of different factors on survival prognosis, the decision curve of a single independent prognostically significant clinical factor and a combination model of clinical factors were constructed using the rmda package in R3.6.1 (Version 1.6) [27].

**Fig. 4:**
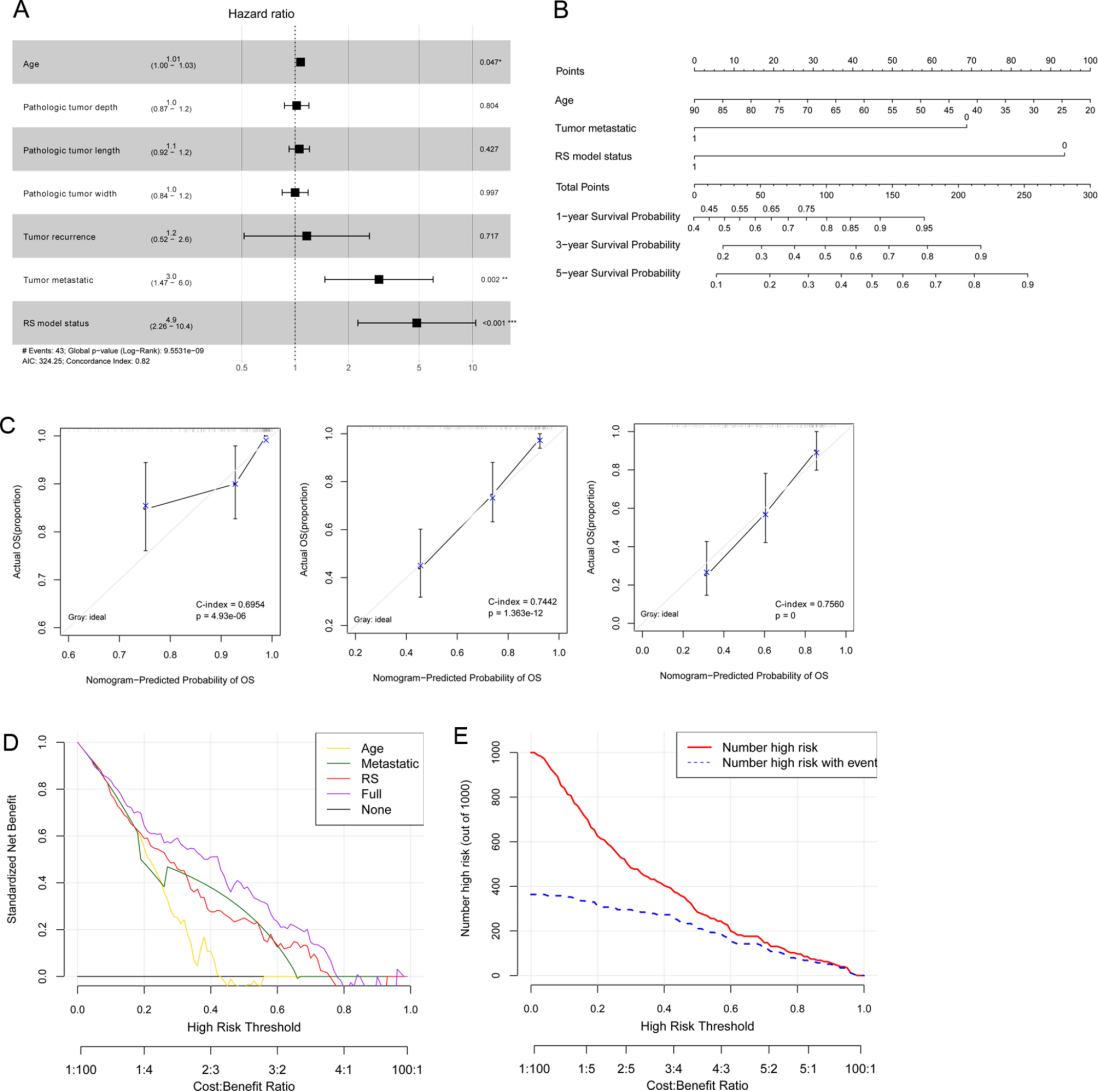
Forest map of prognostic clinical factors, nomogram diagram of independent prognostic factors, and decision line curve. a: Forest map of clinical factors related to prognosis. b: Nomogram diagram of independent prognostic factors in the nomogram survival prediction model. c: Momograph of one-year, three-year and five-year survival rate prediction and actual survival rate. The horizontal axis represents the predicted survival rate, and the vertical axis represents the actual survival rate. d: Decision line curve of independent prognosis-associated clinical factors. e: Decision line curve of the combined model

### Immune related analysis

Various types of immune cells are present in the tumor microenvironment. The proportion of immune cells in the OS samples was analyzed using CIBERSORT, and the distribution of these cells in the two risk groups was also analyzed [28]. Subsequently, the ESTIMATE score, immune score, matrix score, and tumor purity of the OS samples in the TCGA dataset were calculated, and the distribution differences of various scores in the two risk groups were compared using the R3.6.1 estimate package [29]. Finally, we calculated the correlation between the characteristic ERS-related genes in the RS model and the immune cells, and estimated scores with significant differences using the cor function in R3.6.1. CIBERSORT is a tool that is used for predicting the expression matrices of immune cell subtype deconvolution. Differences in the proportion of immune cell types obtained in the previous step were compared using the between-group t-test function in R3.6.1.

**Fig. 5:**
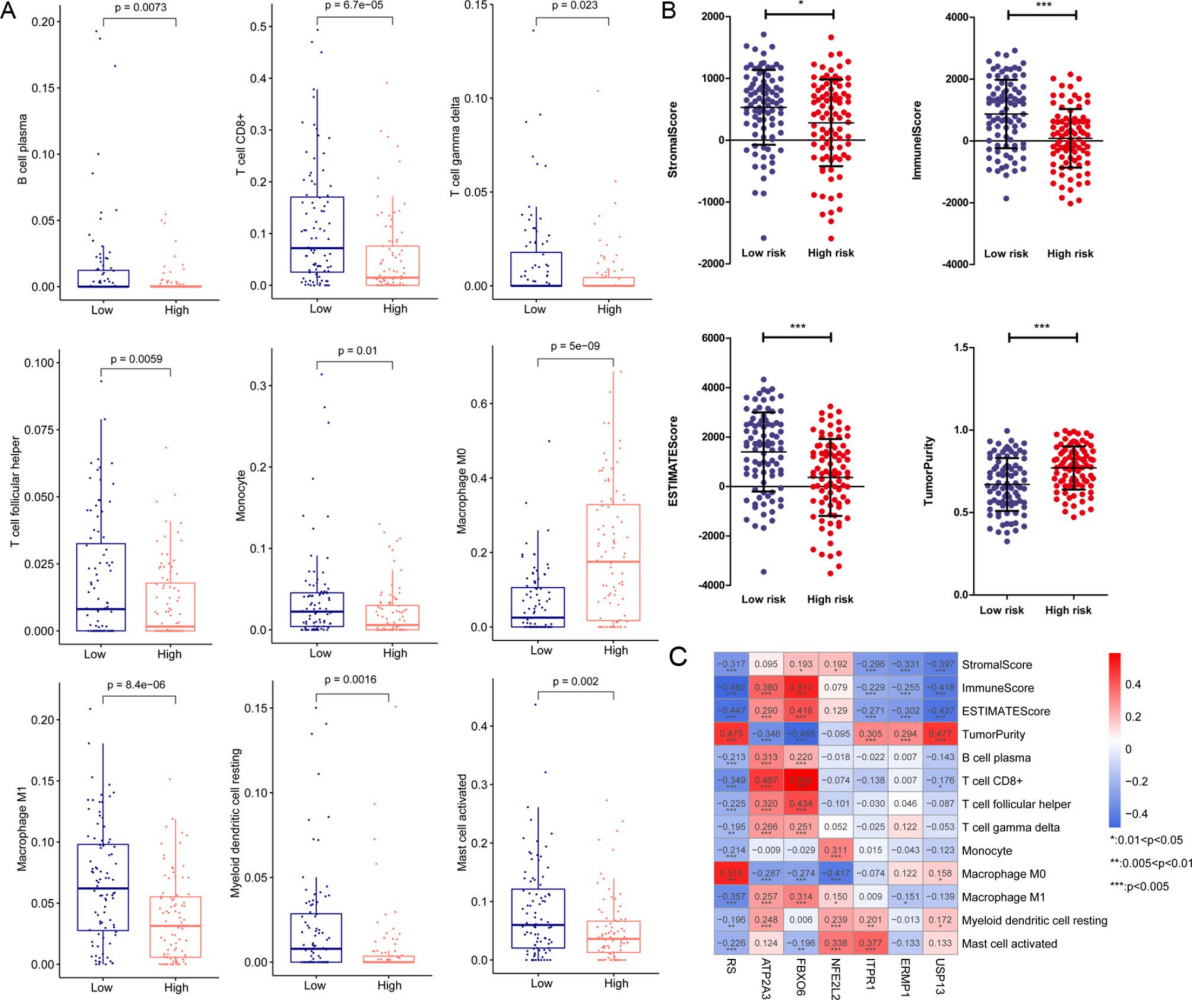
Distribution and potential role of ten immune cells in the high- and low-risk groups. a: Distribution of immune cells in the high- and low- risk groups with significant difference, including plasma cells, CD8 + T cells, follicular helper T cells, gamma delta T cells, monocytes, M0 macrophages, M1 macrophages, resting myeloid dendritic cells, and activated mast cells. b: ESTIMATE score in the high- and low- risk groups. c: The association of ESTIMATE scores and the six survival related ERS-related genes and immune cells distribution in the high- and low- risk groups with significant difference

### KEGG Analysis of genes associated with risk grouping

KEGG data and related gene information was downloaded from the GSEA database [13]. Each KEGG signaling pathway was quantified by the level of gene expression based on the level of whole genome expression of tumor samples in the TCGA dataset using GSVA in R3.6.1 (Version 1.36.3) [30]. Subsequently, the distribution of each quantified pathway in the two risk groups was analyzed using the intergroup t-test in R3.6.1, and FDR < 0.05 was set as the threshold.

KEGG signaling pathways related to the high-risk group were obtained using GSEA [13]. Three key statistical values were included in the GSEA results: nominal P value, normalized enrichment score (NES), and enrichment score. In general, the greater the absolute NES value, the smaller is the significance p or q value, which indicates higher enrichment and reliability of the results. In KEGG analysis, a p-value less than 0.05 as the threshold.

**Fig. 6:**
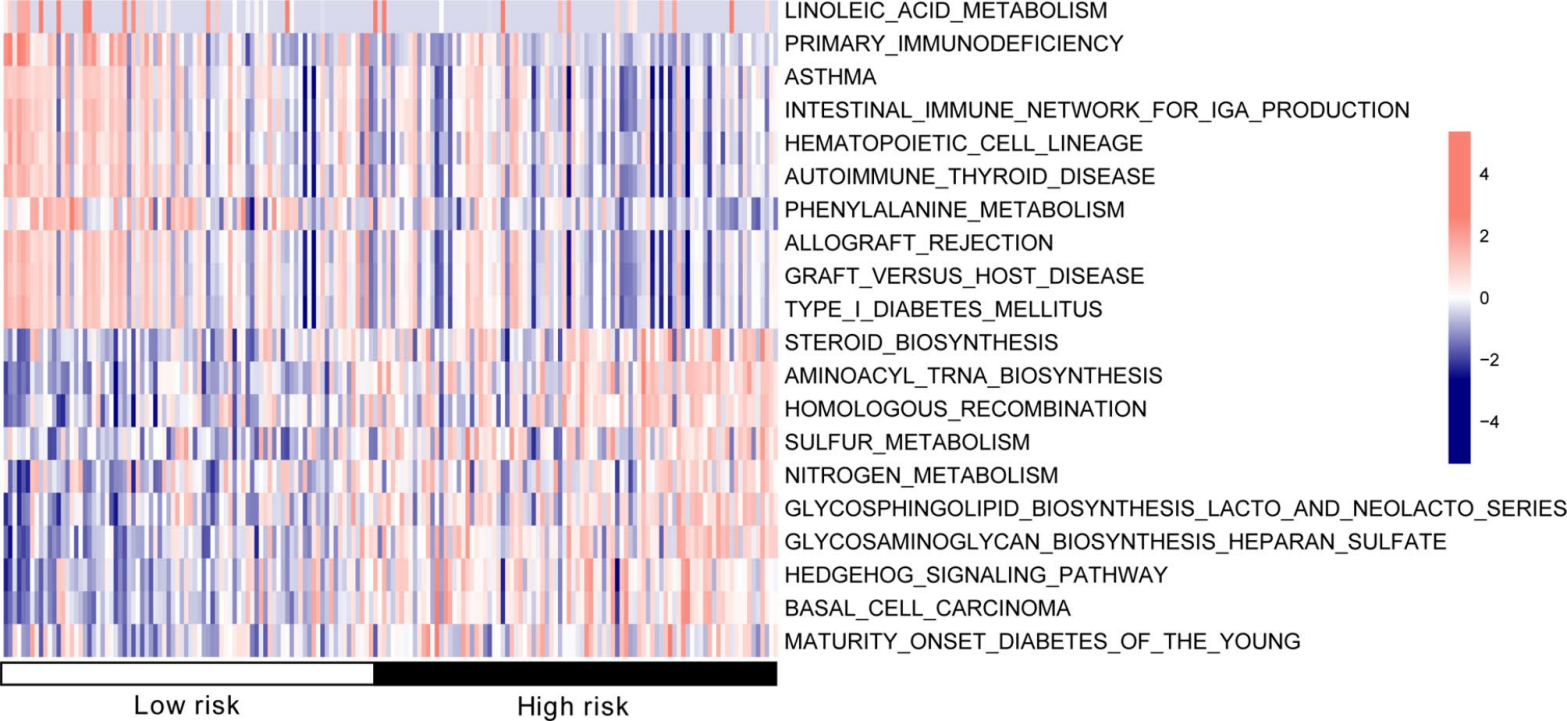
Heatmap of KEGG pathway between the high- and low- risk groups with significant difference

### Real-time quantitative PCR (RT-qPCR)

Six patients with OS with metastasis and six patients without metastasis were recruited from Shengzhou People’s Hospital (Zhejiang, China), and primary tumor tissue samples were collected from each patient. This study was approved by the Ethics Committee of the Shengzhou People’s Hospital (approval no. Sheng Human Medical Ethics 2021, No. 003), and written informed consent was obtained from all participants. All procedures were performed in accordance with the guidelines and regulations of the Declaration of Helsinki.

The expression of six identified ERS-related genes that were significantly associated with prognosis was verified using RT-qPCR. Briefly, total RNA was extracted from tissue samples using the RNAiso Plus kit (Trizol, Takara, Beijing, China) following the manufacturer’s protocol, and was then reverse transcribed into using the PrimeScript™ II 1st Strand cDNA synthesis kit (Takara). The thermal cycling conditions for RT-qPCR were as follows: 50 °C for 3 min, 95 °C for 3 min, a total of 40 cycles at 95 °C for 10 s and 60 °C for 30 s, followed by 95 °C for 15 s, 60 °C for 60 s, and 95 °C for 15 s. The sequences of all primers used in the present study are listed in Table 1. *GAPDH* was used as the housekeeping gene. The relative expression of related genes was calculated using the 2^−ΔΔCt^ method.

**Table 3:** KEGG pathway associated with risk grouping based on GSEA

NAME	SIZE	ES	NES	NOM p-val
KEGG_ADIPOCYTOKINE_SIGNALING_PATHWAY	57	-0.5569825	-1.7915876	0.00193424
KEGG_APOPTOSIS	78	-0.5117746	-1.65334	0.01764706
KEGG_CELL_ADHESION_MOLECULES_CAMS	117	-0.72137684	-1.6764867	0.002
KEGG_CHEMOKINE_SIGNALING_PATHWAY	164	-0.6473055	-1.7237236	0.00197629
KEGG_FATTY_ACID_METABOLISM	33	-0.5824161	-1.618102	0.01734104
KEGG_HISTIDINE_METABOLISM	21	-0.7386318	-1.6555344	0
KEGG_JAK_STAT_SIGNALING_PATHWAY	109	-0.54960704	-1.5821759	0.01171875
KEGG_PEROXISOME	67	-0.4413059	-1.6513382	0.01724138
KEGG_REGULATION_OF_AUTOPHAGY	20	-0.59448904	-1.8016571	0.00386847
KEGG_RIG_I_LIKE_RECEPTOR_SIGNALING_PATHWAY	46	-0.54924124	-1.6602743	0.03281853
KEGG_RNA_POLYMERASE	28	0.5676556	1.7359135	0.0244898
KEGG_T_CELL_RECEPTOR_SIGNALING_PATHWAY	99	-0.595637	-1.6760957	0.03386454
KEGG_TOLL_LIKE_RECEPTOR_SIGNALING_PATHWAY	83	-0.6178474	-1.5817055	0.01629328
KEGG_TRYPTOPHAN_METABOLISM	32	-0.67251796	-1.6750845	0.00389105
KEGG_TYROSINE_METABOLISM	27	-0.7117	-1.6309493	0.003861
KEGG_VASCULAR_SMOOTH_MUSCLE_CONTRACTION	92	-0.6447367	-1.5765048	0.01431493

## Results

### ERS-related DEGs in OS and functional performance

A total of 508 DEGs in metastatic and non-metastatic tissues were identified, which included 367 downregulated and 141 upregulated genes; a volcanic inspection map is shown in Fig. 1A. In total, 256 ERS-related genes were downloaded from the GSEA database. After comparison with the screened DEGs, 41 overlapping genes, i.e., ERS-related DEGs, were identified, including 23 and 18 ERS-related DEGs that were downregulated and upregulated, respectively, in OS with metastasis compared to that in samples without metastasis (Fig. 1B).

The protein–protein interaction network of these 41 ERS-related DEGs was constructed, and 166 pairs of related linkage pairs and 83 co-expression relationship pairs were identified (Fig. 1C).

As shown in Figs. 1D and E and 11 biological processes, such as response to endoplasmic reticulum stress, ubiquitin-dependent ERAD pathway, and endoplasmic reticulum unfolded protein response, as well as eight KEGG pathways, such as pathways of neurodegeneration, multiple diseases, protein processing in endoplasmic reticulum, and thyroid hormone signaling pathway were found to be enriched by the identified ERS-related DEGs.

### Prognostic model construction

In total, 15 ERS-related DEGS that were significantly associated with prognosis were screened, namely *NFE2L2, ATP2A3, LRRK2, BAG6, PIK3R2, FICD, ALOX5, FBXO6, ERMP1, USP13, ITPR1, FAF1, BHLHA15, ERLEC1*, and *UBXN10*. Furthermore, six ERS-related DEGs were identified as independent survival prognostic ERS-related DEGs, namely *FBXO6, ATP2A3, ITPR1, NFE2L2, USP13*, and *ERMP1* (Fig. 2A).

**Fig. 7:**
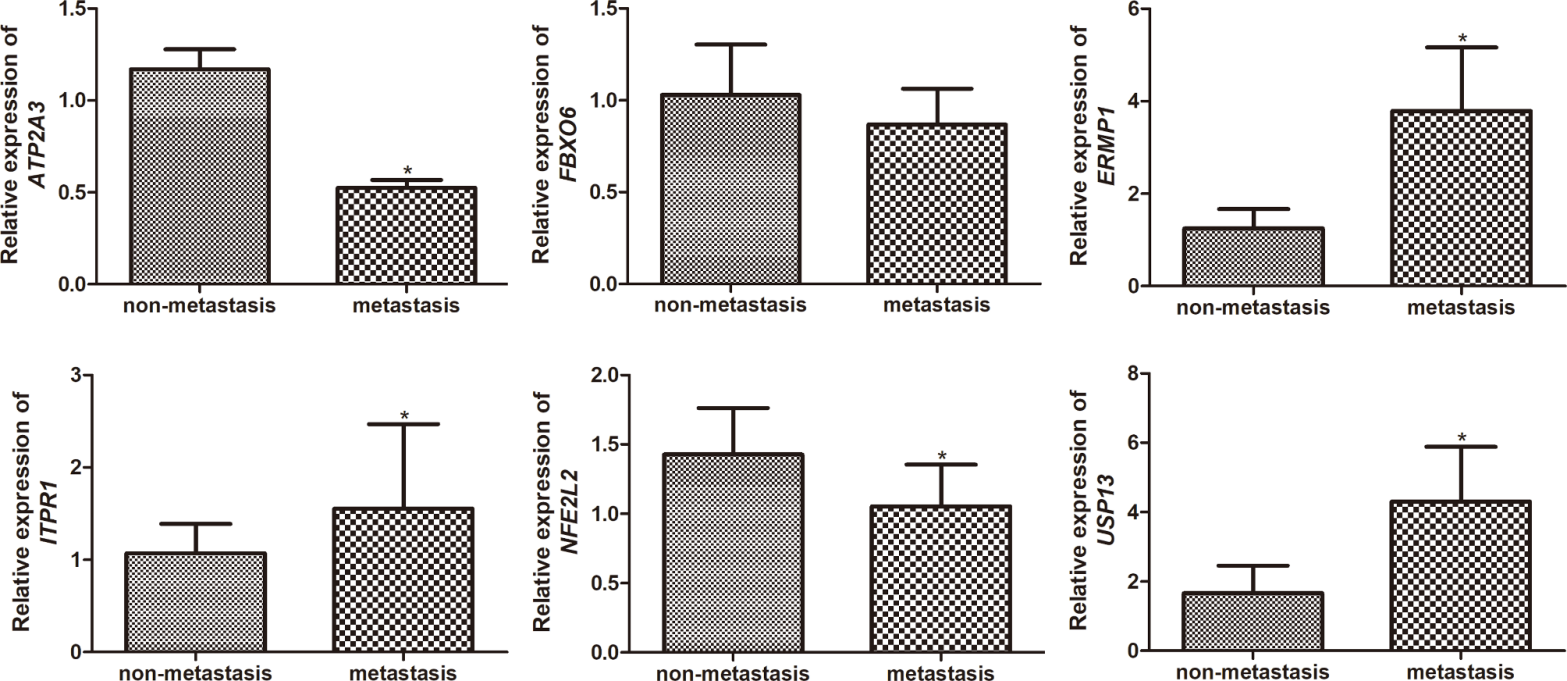
The mRNA expression of six ERS-related prognostic genes in OS patients with or without metastasis. * *P* < 0.05, compared with patients with OS without metastasis

A parameter diagram of the LASSO algorithm is shown in Fig. 2B. Six optimal ERS-related DEGs were confirmed as independent survival prognosis-related genes for patients with OS metastasis. Based on the median expression of these six genes, the samples were categorized into high- and low-expression groups, and the correlation between the level of expression and survival prognosis was analyzed using KM curve analysis, as shown in Fig. 2C. Patients with OS with low expression of *USP13* and *ERMP1* and those with high expression of *FBXO6, ATP2A3, ITPR1*, and *NFE2L2* had better survival.

The RS formula was formulated based on the expression of the six genes and the LASSO regression coefficient, as follows:

RS = (− 0.04799009 × Exp _ATP2A3_) + (0.04418081 × Exp _ERMP1_) + (− 0.08854917 × Exp _FBXO6_) + (− 0.04318914 × Exp _ITPR1_) + (− 0.14873321 × Exp _NFE2L2_) + (0.13858617 × Exp _USP13_).

### Characterized of RS survival and prognosis risk prediction models

In the TCGA training set, low-risk samples stratified by RS values showed better overall survival than high-risk samples (Fig. 3A). The distribution of RS values and survival in the high- and low-risk groups are shown in Fig. 3B. As shown in Fig. 3C, the area under the curve (AUC) of the ROC curve of these genes in the TCGA database was 0.840, with a specificity of 0.869 and a sensitivity of 0.785.

In the GSE39055 dataset, the risk groups that were predicted based on the RS model significantly correlated with the actual prognosis (Fig. 3D). The distribution of RS values and survival in the high- and low-risk groups are shown in Fig. 3E. As shown in Fig. 3F, the AUC of the ROC curve for these genes in the GSE39055 database was 0.789, with a specificity of 0.815 and a sensitivity of 0.800.

### The characterization of the nomogram survival rate model

As shown in Table 2, age, tumor metastasis, and the RS model were independent prognostic clinical factors, and OS was found to be positively correlated with age, tumor metastasis, and the RS model (Fig. 4A).

To further analyze the correlation between age, tumor metastasis, RS model factors, and survival prognosis, a nomogram survival rate model was constructed (Fig. 4B). The nomogram used the “Total points” axis in the first row to synthesize various clinical indicators to predict the survival period of the sample. The consistency between the predicted 1-year, 3-year and 5-year survival rates of the nomogram survival rate model and the actual 1-year, 3-year and 5-year survival rates was analyzed and verified. As shown in Fig. 4C, the predicted 1-year, 3-year and 5-year survival rates were consistent with the actual 1-year, 3-year and 5-year survival rates.

To assess the net earnings rate of age, tumor metastasis, RS models, and a combined model of clinical factors for survival and prognosis, decision curve analysis was performed. Figure 4D and E show the prediction model with three clinical patients that had the highest earnings rate.

### Immune related analysis

The infiltration of nine types of immune cells in the high-risk group was significantly different from that in the low-risk group, namely plasma cells, CD8 + T cells, follicular helper T cells, gamma delta T cells, monocytes, M0 macrophages, M1 macrophage, resting myeloid dendritic cell, and activated mast cell. The distribution of the immune cells is shown in Fig. 5A.

ESTIMATE was used to evaluate the sample scores. As shown in Fig. 5B, high-risk samples had significantly higher ESTIMATE scores and tumor purity and relatively lower stromal and immune scores. RS was found to be significantly positively correlated with tumor purity and M0 macrophages, whereas it was significantly negatively correlated with stromal score, immune score, ESTIMATE score, and the other immune cell types (Fig. 5C). The relationships between the six survival prognostic ERS-related DEGs and the four ESTIMATE scores or the immune cell types are shown in Fig. 5C.

### KEGG analysis of the risk groups

In total, 101 KEGG signaling pathways with significant differences between the high- and low-risk groups were identified, including glycosaminoglycan biosynthesis, heparan sulfate, aminoacyl tRNA biosynthesis, and RNA polymerase. A heatmap of the top 20 KEGG signaling pathways according to the degree of difference is shown in Fig. 6.

Based on the expression in TCGA tumor samples and a *P* < 0.05, 16 KEGG pathways were found to be significantly related with the risk groups (Table 3). Furthermore, we identified nine overlapping KEGG pathways between GSEA and GSVA, including histidine metabolism, cell adhesion molecule cams, tryptophan metabolism, Toll-like receptor signaling pathway, chemokine signaling pathway, RNA polymerase, Toll-like receptor signaling pathway, and tyrosine metabolism.

### Validation of ERS-related DEGs significantly associated with survival prognosis using RT-qPCR

To validate the reliability of the bioinformatics analysis, six ERS-related DEGs that were significantly associated with survival prognosis (*FBXO6, ATP2A3, ITPR1, NFE2L2, USP13*, and *ERMP1*) were verified with RT-qPCR. Compared to patients with OS without metastasis, the expression of *ATP2A3* and *NFE2L2* in patients with OS with metastasis were found to be significantly decreased (*P* < 0.05), whereas the expression of *ITRP1*, *ERMP1*, and *USP13* were markedly increased in patients with OS with metastasis (*P* < 0.05, Fig. 7). These results are consistent with the expression patterns observed in the bioinformatics analysis. However, no significant difference was found in the expression of *FBXO6* between patients with OS with and without metastasis (*P* > 0.05; Fig. 7). Collectively, these results implied that the consistency rate of the RT-qPCR results with those of the bioinformatics analysis was approximately 83.33%, which suggests relatively high reliability of the bioinformatics analysis.

## Discussion

Improving the overall survival for patients with OS is a clinical challenge, although advancement has been made to a certain degree. Appropriate treatment strategies should be supported by accurate diagnosis and staging to combat this challenge. Recently, imaging and biopsies have become the standard diagnostic strategies for OS, and imaging mainly includes magnetic resonance imaging, computed tomography, bone scintigraphy using technetium, and positron emission tomography [31]. On the other hand, molecular diagnosis is not widely used to diagnose OS. In the present study, six survival-related ERS genes were investigated in OS: *ATP2A3, ERMP1, FBXO6, ITPR1, NFE2L2*, and *USP13*. We constructed a RS model based on these genes and demonstrated their promising ability to predict survival. Furthermore, age, tumor metastasis, and the RS model were independent prognostic clinical factors for OS. The predicted nomogram survival model confirmed a similar performance of one, three, and five year-survival rate to the actual survival rates. The distribution of the nine types of immune cells in the high-risk group was found to be significantly different from that in the low-risk group, including that of plasma cells, CD8 + T cells, and follicular helper T cells. Functional analysis showed that these survival prognostic ERS-related genes were significantly enriched in pathways related to amino acid metabolism and membrane signaling.

ERS can cause aggregation of misfolded protein, which consequently results in dysregulation of cell proliferation and apoptosis [32]. Various regulatory and transcription factors can be triggered by an unfolded protein response [33]. In the present study, a survival prediction model based on six ERS-related DEGs including *ATP2A3, ERMP1, FBXO6, ITPR1, NFE2L2*, and *USP13* was established, which were also validated using RT-qPCR. Our RT-qPCR results showed that the expression of *ATP2A3* and *NFE2L2* was upregulated in patients with OS with metastasis, while the expression of *ITRP1*, *ERMP1*, and *USP13* was downregulated, thereby suggesting the relatively high reliability of the bioinformatic analysis. Based on previous studies, it is known that these genes are involved in several cancers with respect to processes, such as leukotriene biosynthesis and cell apoptosis. *ATP2A3* alteration has been reported in various types of tumors, and is involved in the susceptibility to multiple cancers in humans as a consequence of modulation of gene transcription and cell proliferation [34]. Zhang et al. reported that *ATP2A3*, which is upregulated after salinomycin treatment, may be a potential target of salomycin, which can suppress Ca^2+^ release and trigger ERS to play an anticancer role [35]. ERMP1 is widely expressed in cancers, and is an important participant in the unfolded protein response. Huang et al. showed that *ERMP1* knockdown inhibited cell proliferation, thereby suppressing OS metastasis [36]. FBXO, a core component of the E3 ubiquitin ligase family, is important in multiple cellular processes. Researchers have indicated that FBXOs play critical roles in cancer development [37]. *ITPR1* is highly expressed in OS tissues and cells, and overexpression of *ITPR1* can reportedly inhibit OS cell function [38]. The Nfe2l2/Hmox1 signaling pathway is involved in different cellular stress responses, acts wherein it decreases inflammatory factor levels, decreases oxidative damage, and inhibits cell apoptosis, thereby protecting tissues and organs [39]. *USP13* serves as a critical regulator of tumorigenesis by inhibiting tumor growth in vivo and suppressing lactate production, glucose uptake, and cell proliferation in vitro [40]. Taken together, we speculate that *ATP2A3, ERMP1, FBXO6, ITPR1, NFE2L2*, and *USP13* play important roles in the pathogenesis and progression of OS.

Additionally, the influence of the six ERS-related DEGs on the prediction of OS prognosis was further explored by dividing the samples into high- and low-risk groups. We found significantly higher levels of immune cells in the low-risk group than that in the high-risk group, with the exception of M0 macrophages. Previous evidence suggests that prolonged immune responses and the composition of the tumor microenvironment are significant factors that determine OS prognosis [41, 42]. Tan et al. previously reported that the tumor microenvironment was helpful in identifying the signature of immunotherapy-relevant and prognostic genes [43]. Along with our results, it can be inferred that the six ERS-related prognostic genes could be beneficial for predicting OS with or without metastasis and could be important for clinical decision-making and prognostication.

Age, tumor metastasis, and the RS model were independent prognostic clinical factors for OS. Metastasis is a common cause of high mortality rates in various tumors. Additionally, patients aged > 40 years reported have worse survival outcomes than younger patients [44, 45]. Meanwhile, in the present study, RS was significantly correlated with the four ESTIMA scores and all nine immune cell types, indicating that the RS model status had the highest influence on OS prognosis.

However, our study has some limitations. First, all sample information was downloaded from an online dataset, and the clinical background and information were limited. Second, functional annotation was based on resources from bioinformatics analysis, which should be verified using clinical data. Additionally, immune-related genes involved in OS prognosis should be investigated in the future, and the specific roles of the identified ERS-related genes that are closely related to survival prognosis in OS need to be further explored.

## Conclusion

In conclusion, we constructed a prediction model for OS survival prognosis based on six survival prognostic ERS-related DEGs and three independent prognostic factors that may greatly improve OS prognosis. These findings improve our understanding of the roles of ERS-related genes in OS progression and lay the foundation for the use of the identified six ERS-related survival prognostic genes as potential biomarkers to diagnose OS metastasis and potentials targets to develop therapeutic approaches.

## Data Availability

The datasets supporting the conclusions of this article are available in the UCSC Xena (https://xena.ucsc.edu/) repository and NCBI GEO repository [GSE39055 and hyperlink to dataset in https://www.ncbi.nlm.nih.gov/geo/query/acc.cgi?acc=GSE39055].

## List of Abbreviations

OS: Osteosarcoma
ERS: Endoplasmic reticulum stress
DEGs: Differentially expressed genes
RS: Risk score
KM: Kaplan-Meier
ROC: Receiver operating characteristic curve
ER: Endoplasmic reticulum

## References

[1] Barani M, Mukhtar M, Rahdar A, Sargazi S, Pandey S, Kang M. Recent advances in nanotechnology-based diagnosis and treatments of human osteosarcoma. Biosens (Basel) 2021, 11(2). 10.3390/bios11020055 PMC7924594 33672770

[2] Gill J, Gorlick R (2021). Advancing therapy for osteosarcoma. Nat Rev Clin Oncol.

[3] Csala M, Banhegyi G, Benedetti A (2006). Endoplasmic reticulum: a metabolic compartment. FEBS Lett.

[4] Schroder M, Kaufman RJ (2005). ER stress and the unfolded protein response. Mutat Res.

[5] Schroder M, Kaufman RJ (2005). The mammalian unfolded protein response. Annu Rev Biochem.

[6] Shimizu T, Kamel WA, Yamaguchi-Iwai S, Fukuchi Y, Muto A, Saya H (2017). Calcitriol exerts an anti-tumor effect in osteosarcoma by inducing the endoplasmic reticulum stress response. Cancer Sci.

[7] Lin CC, Kuo CL, Lee MH, Lai KC, Lin JP, Yang JS, Yu CS, Lu CC, Chiang JH, Chueh FS (2011). Wogonin triggers apoptosis in human osteosarcoma U-2 OS cells through the endoplasmic reticulum stress, mitochondrial dysfunction and caspase-3-dependent signaling pathways. Int J Oncol.

[8] Zhang L, Wang Y, Zhang L, Xia X, Chao Y, He R, Han C, Zhao W (2019). ZBTB7A, a miR-663a target gene, protects osteosarcoma from endoplasmic reticulum stress-induced apoptosis by suppressing LncRNA GAS5 expression. Cancer Lett.

[9] Zhao A, Zhang Z, Zhou Y, Li X, Li X, Ma B, Zhang Q (2020). beta-elemonic acid inhibits the growth of human osteosarcoma through endoplasmic reticulum (ER) stress-mediated PERK/eIF2alpha/ATF4/CHOP activation and Wnt/beta-catenin signal suppression. Phytomedicine.

[10] Edgar R, Domrachev M, Lash AE (2002). Gene expression Omnibus: NCBI gene expression and hybridization array data repository. Nucleic Acids Res.

[11] Kelly AD, Haibe-Kains B, Janeway KA, Hill KE, Howe E, Goldsmith J, Kurek K, Perez-Atayde AR, Francoeur N, Fan JB (2013). MicroRNA paraffin-based studies in osteosarcoma reveal reproducible independent prognostic profiles at 14q32. Genome Med.

[12] Ritchie ME, Phipson B, Wu D, Hu Y, Law CW, Shi W, Smyth GK (2015). Limma powers differential expression analyses for RNA-sequencing and microarray studies. Nucleic Acids Res.

[13] Subramanian A, Tamayo P, Mootha VK, Mukherjee S, Ebert BL, Gillette MA, Paulovich A, Pomeroy SL, Golub TR, Lander ES (2005). Gene set enrichment analysis: a knowledge-based approach for interpreting genome-wide expression profiles. Proc Natl Acad Sci U S A.

[14] Szklarczyk D, Morris JH, Cook H, Kuhn M, Wyder S, Simonovic M, Santos A, Doncheva NT, Roth A, Bork P (2017). The STRING database in 2017: quality-controlled protein-protein association networks, made broadly accessible. Nucleic Acids Res.

[15] Shannon P, Markiel A, Ozier O, Baliga NS, Wang JT, Ramage D, Amin N, Schwikowski B, Ideker T (2003). Cytoscape: a software environment for integrated models of biomolecular interaction networks. Genome Res.

[16] Kanehisa M, Furumichi M, Sato Y, Kawashima M, Ishiguro-Watanabe M (2023). KEGG for taxonomy-based analysis of pathways and genomes. Nucleic Acids Res.

[17] Kanehisa M, Goto S (2000). KEGG: kyoto encyclopedia of genes and genomes. Nucleic Acids Res.

[18] Kanehisa M (2019). Toward understanding the origin and evolution of cellular organisms. Protein science: a publication of the Protein Society.

[19] Huang da W, Sherman BT, Lempicki RA (2009). Systematic and integrative analysis of large gene lists using DAVID bioinformatics resources. Nat Protoc.

[20] Huang da W, Sherman BT, Lempicki RA (2009). Bioinformatics enrichment tools: paths toward the comprehensive functional analysis of large gene lists. Nucleic Acids Res.

[21] Wang P, Wang Y, Hang B, Zou X, Mao JH (2016). A novel gene expression-based prognostic scoring system to predict survival in gastric cancer. Oncotarget.

[22] Goeman JJ (2010). L1 penalized estimation in the Cox proportional hazards model. Biometrical J Biometrische Z.

[23] Tibshirani R (1997). The lasso method for variable selection in the Cox model. Stat Med.

[24] Harrell FE, Lee KL, Mark DB (1996). Multivariable prognostic models: issues in developing models, evaluating assumptions and adequacy, and measuring and reducing errors. Stat Med.

[25] Shan S, Chen W, Jia JD (2019). Transcriptome analysis revealed a highly connected Gene Module Associated with cirrhosis to Hepatocellular Carcinoma Development. Front Genet.

[26] Mayr A, Schmid M (2014). Boosting the concordance index for survival data–a unified framework to derive and evaluate biomarker combinations. PLoS ONE.

[27] Vickers AJ, Elkin EB (2006). Decision curve analysis: a novel method for evaluating prediction models. Med Decis Making.

[28] Chen B, Khodadoust MS, Liu CL, Newman AM, Alizadeh AA (2018). Profiling Tumor infiltrating Immune cells with CIBERSORT. Methods Mol Biol.

[29] Hu D, Zhou M, Zhu X. Deciphering Immune-Associated Genes to Predict Survival in Clear Cell Renal Cell Cancer. *Biomed Res Int* 2019, 2019:2506843. 10.1155/2019/2506843 PMC6925759 31886185

[30] Ye L, Zhang T, Kang Z, Guo G, Sun Y, Lin K, Huang Q, Shi X, Ni Z, Ding N (2019). Tumor-infiltrating Immune cells Act as a marker for prognosis in Colorectal Cancer. Front Immunol.

[31] Geller DS, Gorlick R (2010). Osteosarcoma: a review of diagnosis, management, and treatment strategies. Clin Adv Hematol Oncol.

[32] Lee CW, Chi MC, Chang TM, Liu JF (2019). Artocarpin induces cell apoptosis in human osteosarcoma cells through endoplasmic reticulum stress and reactive oxygen species. J Cell Physiol.

[33] Ebrahimi N, Saremi J, Ghanaatian M, Yazdani E, Adelian S, Samsami S, Moradi N, Rostami Ravari N, Ahmadi A, Hamblin MR (2022). The role of endoplasmic reticulum stress in the regulation of long noncoding RNAs in cancer. J Cell Physiol.

[34] Themistocleous SC, Yiallouris A, Tsioutis C, Zaravinos A, Johnson EO, Patrikios I (2021). Clinical significance of P-class pumps in cancer. Oncol Lett.

[35] Zhang Y, Li F, Liu L, Jiang H, Hu H, Du X, Ge X, Cao J, Wang Y (2019). Salinomycin triggers endoplasmic reticulum stress through ATP2A3 upregulation in PC-3 cells. BMC Cancer.

[36] Huang Z, Lan T, Wang J, Chen Z, Zhang X (2021). Identification and validation of seven RNA binding protein genes as a prognostic signature in oral cavity squamous cell carcinoma. Bioengineered.

[37] Liu Y, Pan B, Qu W, Cao Y, Li J, Zhao H (2021). Systematic analysis of the expression and prognosis relevance of FBXO family reveals the significance of FBXO1 in human breast cancer. Cancer Cell Int.

[38] Yu M, Lu W, Cao Z, Xuan T (2021). LncRNA LINC00662 exerts an oncogenic effect on Osteosarcoma by the miR-16-5p/ITPR1 Axis. J Oncol.

[39] Zheng T, Huang Z, Ling H, Li J, Cheng H, Chen D, Lu Q, Zhao J, Su W. The mechanism of the Nfe2l2/Hmox1 signaling pathway in ferroptosis regulation in acute compartment syndrome. J Biochem Mol Toxicol 2022:e23228. 10.1002/jbt.23228 PMC10078270 36193742

[40] Qu Z, Zhang R, Su M, Liu W (2019). USP13 serves as a tumor suppressor via the PTEN/AKT pathway in oral squamous cell carcinoma. Cancer Manag Res.

[41] Shu Y, Peng J, Feng Z, Hu K, Li T, Zhu P, Cheng T, Hao L (2022). Osteosarcoma subtypes based on platelet-related genes and tumor microenvironment characteristics. Front Oncol.

[42] Yao S, Deng M, Du X, Chen Q, Huang R (2022). Identification of two Novel Immune Subtypes characterized by distinct prognosis and Tumor Microenvironment in Osteosarcoma. J Immunol Res.

[43] Tan J, Feng X, Wu H, Yang B, Shi M, Xie C, Su Z, Li L, Luo M, Zuo Z (2022). Characterization of the Tumor Microenvironment in Osteosarcoma identifies prognostic- and immunotherapy-relevant Gene Signatures. J Immunol Res.

[44] Sadykova LR, Ntekim AI, Muyangwa-Semenova M, Rutland CS, Jeyapalan JN, Blatt N, Rizvanov AA (2020). Epidemiology and risk factors of Osteosarcoma. Cancer Invest.

[45] Harting MT, Lally KP, Andrassy RJ, Vaporciyan AA, Cox CS, Hayes-Jordan A, Blakely ML (2010). Age as a prognostic factor for patients with osteosarcoma: an analysis of 438 patients. J Cancer Res Clin Oncol.

